# Metabolic Dysfunction–Associated Steatotic Liver Disease and Respiratory Disorders: A Systematic Review of Clinical and Pathophysiological Associations

**DOI:** 10.1007/s13679-026-00713-8

**Published:** 2026-04-17

**Authors:** Vasiliki Epameinondas Georgakopoulou, Paschalis Steiropoulos, Theodoros Androutsakos, Irena Karabella, Konstantinos Reppas, Evangelos Cholongitas, Maria Dalamaga

**Affiliations:** 1 Department of Pathophysiology, Laiko General Hospital, National and Kapodistrian University of Athens, Athens, 11527 Greece; 2 Department of Pneumonology, Medical School, Democritus University of Thrace, Alexandroupolis, 68100 Greece; 3 Department of Internal Medicine, Sotiria Thoracic Diseases General Hospital, Athens, 11527 Greece; 4 First Department of Internal Medicine, Laiko General Hospital, National and Kapodistrian University of Athens, Athens, 11527 Greece; 5 Department of Biologic Chemistry, School of Medicine, National and Kapodistrian University of Athens, 75 Mikras Asias St., Athens, Goudi, 115 27 Greece

**Keywords:** Asthma, Chronic obstructive pulmonary disease, Fibrosis, Hypoxia, Interstitial lung disease, Liver–lung axis, Metabolic dysfunction–associated steatotic liver disease, MASLD, NAFLD, Obesity, Obstructive Sleep Apnea, Pulmonary hypertension, Respiratory disorders

## Abstract

**Purpose of Review:**

Metabolic dysfunction–associated steatotic liver disease (MASLD) represents the hepatic manifestation of ectopic fat accumulation and adipose tissue dysfunction. Excess visceral adiposity promotes adipose tissue hypoxia, macrophage infiltration, and chronic low-grade systemic inflammation, generating systemic metabolic stress that may extend to the lung through a proposed liver–lung axis (hepatopulmonary crosstalk). This systematic review synthesizes current evidence on the associations between MASLD and major respiratory diseases, with emphasis on shared obesity-driven mechanisms. A systematic review was conducted in accordance with the PRISMA 2020 guidelines and registered in PROSPERO (CRD420261301893). PubMed/MEDLINE, Embase, Web of Science, and Google Scholar were searched from inception through February 28, 2026.

**Recent Findings:**

Twenty-two studies, 21 observational and 1 Mendelian randomization study (MRS) study reported associations between MASLD and several respiratory conditions, including chronic obstructive pulmonary disease (COPD), asthma, obstructive sleep apnea (OSA), interstitial lung disease, pulmonary hypertension, and respiratory mortality. MASLD prevalence was high among patients with COPD and OSA, and was associated with worse respiratory phenotypes, increased exacerbations, and higher respiratory mortality in several cohorts. Liver fibrosis appeared more strongly associated with impaired lung function and adverse outcomes than steatosis alone. MRS were critically re-evaluated, and do not consistently support causal relationships for most respiratory conditions, with the exception of OSA, which showed evidence suggestive of a potential causal association. Shared mechanisms include visceral adiposity, adipokine imbalance, insulin resistance, systemic cytokine activation (IL-6, TNF-α), endothelial dysfunction, oxidative stress, intermittent hypoxia, and profibrotic transforming growth factor-beta (TGF-β)–mediated pathways.

**Summary:**

Current evidence supports a clinically significant association between MASLD and respiratory disease, particularly COPD, OSA and asthma. Recognizing MASLD as a marker of systemic metabolic dysfunction may improve integrated cardiometabolic and pulmonary risk stratification. Prospective and interventional studies targeting weight reduction and metabolic signaling are needed to clarify causality and therapeutic implications.

**Supplementary Information:**

The online version contains supplementary material available at 10.1007/s13679-026-00713-8.

## Introduction

Metabolic dysfunction–associated steatotic liver disease (MASLD), previously termed non-alcoholic fatty liver disease (NAFLD), represents the hepatic manifestation of systemic metabolic dysfunction and is currently the most prevalent chronic liver disease, affecting approximately 30–40% of the adult population globally, with regional variation ranging from 25.1% in Western Europe to 44.4% in Latin America [1–3]. The 2023 international consensus-led nomenclature change from NAFLD to MASLD reflects a shift toward a positive, metabolically grounded definition of disease, requiring evidence of hepatic steatosis in the presence of cardiometabolic risk factors. This transition was intended to better capture the underlying pathophysiology, improve patient stratification, and eliminate the exclusionary and potentially stigmatizing elements embedded in the prior terminology. The concordance between NAFLD and MASLD definitions is approximately 99%, validating research continuity. In most patients, this dysfunction arises from excess visceral adiposity and ectopic fat deposition, with MASLD affecting 70–80% of individuals with obesity [4]. However, lean MASLD (Body Mass Index (BMI) < 25 kg/m² or < 23 kg/m² in Asians) affects approximately 6–9% of lean adults and exhibits similar or higher liver-related mortality despite lower cardiovascular risk compared to non-lean MASLD [5, 6]. Beyond its classical hepatic complications, namely steatohepatitis, progressive fibrosis, cirrhosis, and hepatocellular carcinoma, MASLD is now understood as a systemic metabolic disorder with far-reaching clinical implications [6, 7]. Given that obesity-associated adipose tissue functions as an active endocrine organ, secreting proinflammatory cytokines and adipokines, MASLD may serve as a measurable indicator of systemic metabolic inflammation rather than an isolated hepatic condition [8].

Accumulating evidence over the past decade has established MASLD as a multisystem disease extending well beyond the liver. Cardiovascular disease and extrahepatic cancer are now recognized as the leading causes of mortality in non-cirrhotic MASLD patients, with strong associations reported between hepatic steatosis and atherosclerosis, heart failure, and arrhythmias [7]. However, in patients with MASLD-related cirrhosis, liver-related deaths (10-year cumulative incidence 9.2%) and cardiovascular deaths (10-year cumulative incidence 17.3%) become co-dominant causes of mortality [9]. In parallel, MASLD has been linked to chronic kidney disease, endocrine dysfunction, and adverse metabolic outcomes independent of traditional risk factors [6]. More recently, attention has shifted toward the respiratory system, as epidemiological and mechanistic data increasingly suggest that pulmonary diseases may represent another clinically associated extrahepatic manifestation of MASLD.

Emerging evidence suggests biological communication between the liver and the lung. Shared pathophysiological pathways, including visceral obesity, chronic low-grade systemic inflammation, adipokine imbalance, insulin resistance, oxidative stress, endothelial dysfunction, and altered adipokine signaling, provide a mechanistic model linking hepatic steatosis to pulmonary pathology [10, 11]. Furthermore, hypoxia-related mechanisms, particularly intermittent hypoxia, have been proposed as key modulators within this interorgan crosstalk. Intermittent hypoxia may exacerbate hepatic lipid accumulation and fibrogenesis, while systemic inflammation originating from hepatic metabolic dysfunction may contribute to airway remodeling, impaired lung function, and vascular alterations in the pulmonary circulation [12, 13]. This reciprocal interaction suggests that liver and respiratory diseases may not merely coexist but may biologically influence and potentially amplify one another.

Observational studies have reported an increased prevalence and worse outcomes of MASLD among patients with chronic obstructive pulmonary disease (COPD), as well as associations with asthma and obstructive sleep apnea (OSA), the latter being supported by strong mechanistic and clinical evidence [13–16]. Emerging data also suggest potential links with interstitial lung diseases, pulmonary hypertension, pulmonary embolism and other chronic respiratory disorders, although findings remain inconsistent and methodologically heterogeneous [17, 18]. Evidence regarding non–cystic fibrosis bronchiectasis is particularly limited.

Despite the expanding body of research, current knowledge is limited across disease-specific investigations, often employing heterogeneous diagnostic criteria for both liver and respiratory conditions and showing substantial variability in confounder adjustment, particularly for obesity and metabolic syndrome components. To date, no comprehensive systematic review has synthesized the available evidence across the full spectrum of major respiratory diseases while critically addressing methodological differences and metabolic confounding.

Given the multisystem nature of MASLD and the shared metabolic and inflammatory pathways underlying diverse pulmonary conditions, the present review adopts a broad integrative approach across respiratory phenotypes rather than a disease-specific framework. This strategy aims to capture the systemic nature of liver–lung interactions, acknowledging that MASLD may act as a common pathophysiological substrate influencing multiple respiratory outcomes.

Therefore, the aim of the present systematic review is to systematically synthesize and integrate the existing evidence on the association between MASLD and major respiratory diseases, including COPD, asthma, OSA, idiopathic pulmonary fibrosis (IPF), in adult populations, with particular emphasis on shared pathophysiological mechanisms, the concept of a liver–lung axis and methodological heterogeneity.

## Materials and Methods

### Study Design and Reporting Standards

This systematic review was conducted in accordance with the Preferred Reporting Items for Systematic Reviews and Meta-Analyses (PRISMA) 2020 guidelines [19]. The review protocol was updated prior to study selection to predefine eligibility criteria, outcomes of interest, subgroup analyses and methods for qualitative and quantitative synthesis. The review protocol was registered on the International Prospective Register of Systematic Reviews (PROSPERO) to enhance transparency and minimize the risk of duplication (ID number CRD420261301893).

### Eligibility Criteria

Eligible studies were original human investigations examining the association between MASLD, including studies using the terms NAFLD or MAFLD, and one or more predefined respiratory diseases in adult populations. Respiratory outcomes of interest included COPD, asthma, OSA, interstitial lung disease (including IPF, pulmonary hypertension, and bronchiectasis.

Observational study designs were eligible, including cross-sectional studies, case–control studies and prospective or retrospective cohort studies. Mendelian randomization (MR) studies were also eligible. Studies were required to report a clear definition of hepatic steatosis, diagnosed using imaging modalities [ultrasound, computed tomography (CT), magnetic resonance imaging, magnetic resonance proton density fat fraction, vibration-controlled transient elastography with controlled attenuation parameter (CAP)], liver biopsy, or validated serum-based non-invasive indices [e.g. fatty liver index (FLI), hepatic steatosis index (HIS), NAFLD fibrosis score (NFS), Fibrosis-4 (FIB-4) index, the Aspartate Aminotransferase to Platelet Ratio Index (APRI) score, the Forns index, the Enhanced Liver Fibrosis (ELF) test, the FibroScan-AST (FAST) score, and the Agile-3 + score. Studies assessing liver fibrosis using liver stiffness measurement by transient elastography or magnetic resonance elastography were also eligible. Respiratory diseases had to be defined using established clinical criteria, physician diagnosis, validated questionnaires, spirometric criteria [post-bronchodilator forced expiratory volume in one second/forced vital capacity (FEV1/FVC) < 0.7 for COPD], polysomnography (apnea–hypopnea index (AHI) ≥ 5 events/hour for OSA, home sleep apnea testing, imaging findings, or diagnostic coding systems.

Studies involving pediatric populations, animal or in vitro experiments, case reports, case series, editorials, narrative reviews and conference abstracts without full data were excluded. Studies focusing exclusively on cystic fibrosis–related lung disease were also excluded. No restrictions were applied regarding publication year. Only articles published in English were considered.

### Information Sources and Search Strategy

A comprehensive literature search was performed in PubMed/MEDLINE, Embase, and Web of Science from inception to February 28, 2026. In addition, Google Scholar was used as a supplementary source to identify potentially relevant studies not captured in the primary databases. Given its limited reproducibility, Google Scholar was not used as a primary systematic database. Instead, the first several hundred results sorted by relevance were screened, and citation tracking was performed to identify additional eligible studies.

The search strategy combined Medical Subject Headings (MeSH) terms, controlled vocabulary terms (Emtree for Embase), and free-text terms related to steatotic liver disease and respiratory disorders. Core liver-related terms included “NAFLD”, “non-alcoholic fatty liver disease”, “MASLD”, “metabolic dysfunction-associated steatotic liver disease”, “MAFLD”, “metabolic-associated fatty liver disease”, “fatty liver”, “steatotic liver disease”, “steatohepatitis”, “NASH”, and “hepatic steatosis”. These were combined using the Boolean operator “AND” with respiratory disease terms specific to each disease category, including “COPD”, “chronic obstructive pulmonary disease”, “airflow obstruction”, “asthma”, “obstructive sleep apnea”, “OSA”, “sleep-disordered breathing”, “sleep apnea syndrome”, “interstitial lung disease”, “pulmonary fibrosis”, “idiopathic pulmonary fibrosis”, “pulmonary hypertension”, “pulmonary embolism and “bronchiectasis”.

Search strategies were adapted for each database to optimize disease-specific sensitivity. The full electronic search strategy for at least one database is provided in Supplementary Material (Table S1). Reference lists of eligible articles and relevant review articles were manually screened to identify additional studies. Forward citation searching was performed using Web of Science for key included studies. Duplicate records were removed using EndNote (Clarivate Analytics) prior to screening.

### Study Selection

A total of 1,450 records were identified through database searching (PubMed/MEDLINE: 412; Embase: 538; Web of Science: 286; Google Scholar: 214). An additional 18 records were identified through manual screening of reference lists and citation tracking.

After removal of 438 duplicate records, 1,030 unique articles remained and were subjected to title and abstract screening. Two reviewers (VEG and EC) independently screened titles and abstracts. Disagreements were resolved by consensus or consultation with a third reviewer (MD).

Of these, 930 records were excluded as clearly irrelevant to the research question, primarily due to unrelated outcomes, ineligible populations, or non-original article types. One hundred full-text articles were retrieved and assessed for eligibility. Full-text screening was performed independently by two reviewers, with disagreements resolved through discussion or adjudication by a third reviewer. Following detailed evaluation, 78 studies were excluded for the following reasons: absence of eligible respiratory outcomes (*n* = 18); lack of appropriate liver disease definition or exposure characterization (*n* = 14); ineligible study population (*n* = 15); inappropriate publication type (reviews, editorials, letters, conference abstracts, or case reports; *n* = 12); insufficient data for extraction or absence of extractable outcome measures (*n* = 7); non-English language (*n* = 6); and inability to retrieve full text (*n* = 6).

**Fig. 1:**
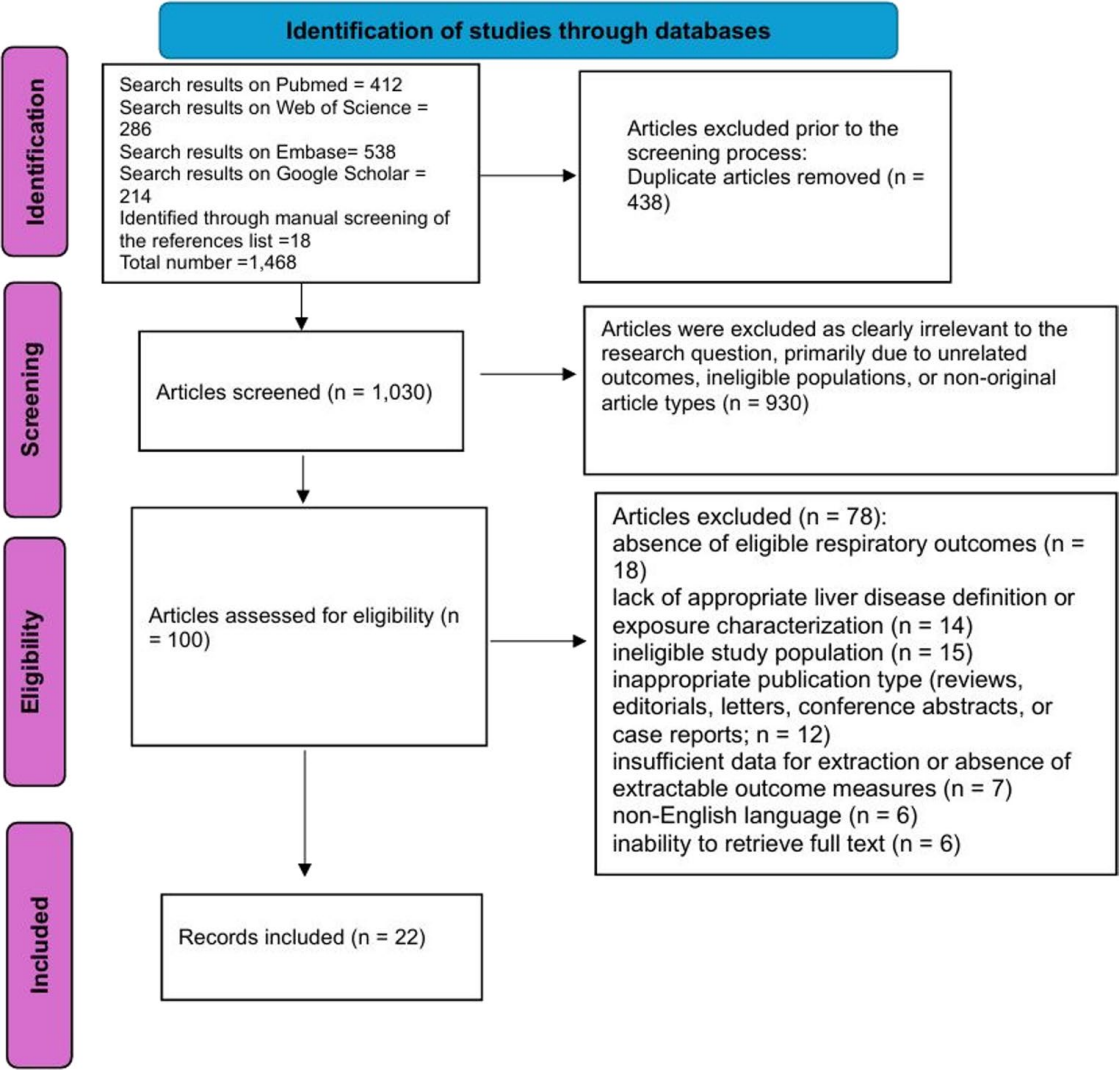
Flowchart of the study selection process

Ultimately, 22 studies met the predefined inclusion criteria and were included in the qualitative synthesis. No studies were included in quantitative synthesis (meta-analysis) due to substantial clinical and methodological heterogeneity. Figure 1 illustrates the study selection process in accordance with the PRISMA 2020 flow diagram.

### Data Extraction

Data were independently extracted by two reviewers (VEG and EC) from each included study using a predefined and piloted extraction form. For each study, the following information was collected: first author and year of publication, country, study design, study setting, population characteristics and respiratory disease phenotype, sample size, method used to assess hepatic steatosis, prevalence of steatotic liver disease, the main reported association between hepatic steatosis and respiratory outcomes, effect estimates with 95% confidence intervals (CI), and variables included in adjusted analyses. Where applicable, information on liver disease severity (e.g. fibrosis or cirrhosis) and study-specific definitions of NAFLD, MAFLD, or MASLD were also captured to facilitate comparison across studies. Funding sources and potential conflicts of interest were also recorded. Any discrepancies in data extraction were resolved through discussion. The data extraction form is provided in Supplementary Material (Table S2).

### Risk of Bias Assessment

Risk of bias was assessed according to study design using validated appraisal tools. Cohort and case-control studies were evaluated using the Newcastle–Ottawa Scale (NOS) [20], which examines selection of study groups, comparability of cohorts, and ascertainment of outcomes. Studies were rated as having low (7–9 stars), moderate (4–6 stars), or high (0–3 stars) risk of bias. Cross-sectional studies were assessed using the Joanna Briggs Institute (JBI) Critical Appraisal Checklist for Analytical Cross-Sectional Studies [21], focusing on participant selection, measurement of exposure and outcomes, identification and control of confounding factors, and appropriateness of statistical analyses. MRS were assessed using the STROBE-MR checklist or relevant quality criteria for genetic epidemiological studies. Each study was independently appraised by two reviewers (VEG and EC), and discrepancies in assessments were resolved through discussion. The results of the risk of bias assessment are presented in the Supplementary File 1. Risk of bias findings were considered when interpreting the strength of evidence for each respiratory disease category.

### Data Synthesis

Given the substantial clinical and methodological heterogeneity across studies, including differences in respiratory phenotypes (COPD, asthma, OSA, pulmonary hypertension, pulmonary embolism and broader respiratory outcomes), definitions of steatotic liver disease (NAFLD, MAFLD, MASLD), assessment methods (serum indices, imaging, elastography, biopsy), and outcome measures (prevalence, incidence, severity, and prognosis), a quantitative meta-analysis was not considered appropriate. Therefore, a narrative synthesis was performed following the Synthesis Without Meta-analysis (SWiM) reporting guidelines.

In particular, the decision not to perform a quantitative meta-analysis was based on substantial heterogeneity across the included studies at multiple levels. Clinical heterogeneity was evident in the wide range of respiratory phenotypes and differences in study populations, while methodological heterogeneity arose from variability in study design, diagnostic definitions of MASLD/NAFLD/MAFLD, and assessment methods for both hepatic and respiratory outcomes. Furthermore, outcome heterogeneity was considerable, with studies reporting diverse endpoints, including prevalence, incidence, lung function parameters, exacerbations, fibrosis severity, and mortality. Effect estimates were not sufficiently comparable to permit meaningful pooling, due to differences in exposure definitions, outcome measurements, covariate adjustment strategies, and reporting of effect measures (e.g. odds ratios, hazard ratios, and regression coefficients). Although subgroup meta-analyses were considered for individual respiratory disease categories, these were not performed due to the limited number of studies within each subgroup and the persistence of substantial heterogeneity even within disease-specific groups. Therefore, a structured narrative synthesis, supported by grouping of studies according to respiratory phenotype and study design, was deemed the most appropriate methodological approach.

An additional methodological consideration relates to the role of metabolic variables included in multivariable adjustment models. Factors such as body mass index, insulin resistance, diabetes mellitus, and lipid parameters may act not only as confounders but also as intermediates within the causal pathway linking MASLD to respiratory outcomes. As a result, adjustment for these variables may have heterogeneous effects across studies. While controlling for such factors may reduce confounding by shared cardiometabolic risk, it may also lead to over-adjustment by attenuating biologically meaningful pathways through which MASLD exerts systemic effects. This is particularly relevant given that MASLD itself reflects a state of metabolic dysfunction closely intertwined with these variables. Consequently, studies employing extensive metabolic adjustment may underestimate true associations, whereas those with limited adjustment may be more susceptible to residual confounding. Therefore, the variability in covariate selection and modeling strategies across studies represents an additional source of methodological heterogeneity and may partly explain inconsistencies in reported effect estimates.

Results were synthesized by grouping studies according to the primary respiratory condition evaluated and by distinguishing between cross-sectional and longitudinal designs. Within each group, findings were compared with respect to the direction and magnitude of associations, the degree of statistical adjustment for metabolic and lifestyle confounders, and evidence of dose–response or nonlinear relationships. Particular emphasis was placed on differentiating associations related to hepatic steatosis from those related to more advanced liver disease (fibrosis or cirrhosis), when reported. Discrepancies between studies were explored in relation to study design, population characteristics, steatosis definition, and adjustment strategies.

The certainty of evidence for each respiratory disease category was assessed using the Grading of Recommendations Assessment, Development and Evaluation (GRADE) approach, considering risk of bias, inconsistency, indirectness, imprecision, and publication bias. Vote counting based on the direction of effect was used to summarize findings when quantitative pooling was not feasible, with results presented in summary tables.

Given the observational nature of most included studies, directionality and causality were not assumed, and findings were interpreted as associations unless supported by longitudinal or genetic evidence.

## Results

### MASLD and the Clinical Spectrum of Respiratory Diseases

A total of 22 studies were included [14–18, 22–38]. Overall, the studies summarized in Table 1 consistently show that steatotic liver disease (including NAFLD, MAFLD, and MASLD, as originally defined in each study) is closely linked with a broad range of respiratory disorders, through epidemiological associations, functional impairment, and adverse clinical outcomes. Importantly, these associations are observed across diverse populations, ranging from highly selected clinical cohorts to large nationwide population-based databases. No eligible studies specifically evaluating the association between MASLD and non–cystic fibrosis bronchiectasis were identified, despite its inclusion in the predefined eligibility criteria.

**Table 1:** Summary of the characteristics of the included studies

Associations of MASLD with Respiratory Outcomes							
Author (Year)	Country/Setting	Study Design	Population (*N*)	Steatosis Assessment	Prevalence	Key Findings	Adjusted Variables
Benotti et al., 2016 [33]	USA/Geisinger Obesity Institute bariatric surgery program	Retrospective cohort	Adults with severe obesity undergoing bariatric surgery; OSA; *N* = 362	Intraoperative liver biopsy; steatosis, lobular inflammation, and fibrosis assessed histologically using NASH CRN criteria	44% steatosis; 38% inflammation and/or fibrosis; 18% normal histology	OSA severity (AHI) and hypoxia parameters were positively associated with NAFLD severity only in patients without metabolic syndrome; no significant association in patients with metabolic syndrome	Age, sex, BMI
Viglino et al., 2017 [14]	France/Grenoble University Hospital outpatient COPD cohorts	Cross-sectional observational analysis	COPD (GOLD I–IV, mainly GOLD I–II); *N* = 111	FibroMax SteatoTest; steatosis defined as ≥S2	41.4% overall; 11.8% in GOLD I, 47.0% in GOLD II, 46.4% in GOLD III–IV	Steatosis was independently associated with COPD severity: GOLD II vs. I OR 21.80 (95% CI 2.36–201.02); GOLD III–IV vs. I OR 26.77 (95% CI 2.34–306.29)	Age, sex, BMI
Viglino et al., 2018 [15]	France/Grenoble Alpes University Hospital	Prospective cohort with 5-year follow-up	COPD (FEV1/FVC < 70%; GOLD I–IV); *N* = 111	FibroMax SteatoTest; steatosis defined as ≥S2	41% (46/111)	At baseline, COPD severity did not differ significantly between patients with and without steatosis; FEV1 (%) and FEV1/FVC were also comparable, suggesting no independent baseline association between steatosis and lung function	Age, BMI, sex, inhaled corticosteroids, dyslipidemia, diabetes
Lee et al., 2020 [25]	South Korea/Seoul National University Boramae Medical Center	Cross-sectional analysis within a prospective biopsy-proven NAFLD cohort	Adults with biopsy-proven NAFLD; chronic lung disease requiring treatment excluded; *N* = 420	Liver biopsy; steatosis graded S1–S3, fibrosis F0–F4; advanced fibrosis ≥F3	Not reported as a binary prevalence in summary	Steatosis severity was associated with lower FVC (%) and higher FEV1/FVC in unadjusted and partially adjusted models, but the FVC association attenuated after full adjustment. Fibrosis severity remained independently associated with reduced post-bronchodilator FVC (%): β = −4.41 per fibrosis stage (95% CI − 8.39 to − 0.43; *p* = 0.031)	Age ≥ 65 years, sex, smoking, COPD, hs-CRP, appendicular skeletal muscle mass, visceral adipose tissue area, diabetes mellitus
Chung et al., 2021 [17]	South Korea/Korean NHIS screening cohort, 2009–2015	Nationwide prospective cohort	Adults ≥ 20 years without OSA at baseline; incident OSA; *N* = 8,116,524	FLI; NAFLD defined as FLI ≥ 60	11.5% with FLI ≥ 60; 22.6% with FLI 30–60	NAFLD was independently associated with increased incident OSA in a dose-dependent manner: FLI 30–60 vs. < 30 aHR 1.15 (95% CI 1.12–1.18); FLI ≥ 60 vs. < 30 aHR 1.21 (95% CI 1.17–1.26). Associations persisted across BMI and abdominal obesity strata	Age, sex, smoking, alcohol, exercise, income, hypertension, dyslipidemia, diabetes, BMI; alternative models adjusted for waist circumference or lipid accumulation product instead of BMI
Roh et al., 2022 [16]	South Korea/NHIS-NSC, 2009–2014	Nationwide retrospective cohort	Adults ≥ 20 years without prior asthma, smoking history, or major cardiometabolic comorbidity; incident adult-onset asthma; *N* = 160,603	FLI (< 30, 30–<60, ≥ 60) and HSI ≥ 36	Asthma incidence: 10.1% in FLI < 30, 10.8% in FLI 30–<60, 10.5% in FLI ≥ 60; 11.1% in HSI ≥ 36 vs. 10.1% in HSI < 36	NAFLD was independently associated with asthma incidence: highest vs. lowest FLI HR 1.25 (95% CI 1.15–1.36); HSI-defined steatosis HR 1.12 (95% CI 1.07–1.17). Association was stronger in women (HR 1.46)	Age, sex, physical activity, alcohol, systolic BP, diastolic BP, fasting blood glucose, LDL-cholesterol
Türker et al., 2022 [27]	Turkey/Florence Nightingale Hospital, Istanbul	Single-center retrospective observational study	Adults with NASH-related cirrhosis; outcome was portopulmonary hypertension (PoPH); *N* = 171, including 43 with PoPH	NASH cirrhosis diagnosed clinically and radiologically (US/CT); liver biopsy contraindicated	All participants had advanced steatotic liver disease; PoPH prevalence 25.1%	PoPH was associated with higher BMI, MELD score, bilirubin, triglycerides, and TSH and lower hemoglobin. In multivariable analysis, TSH was the only independent predictor: OR 1.18 (95% CI 1.01–1.43)	Age, MELD score, BMI, hemoglobin, total bilirubin, TSH, triglycerides
Feng et al., 2024 [30]	USA/NHANES 2007–2012 + two-sample bidirectional MR	Cross-sectional population-based analysis + MR	Adults ≥ 18 years with spirometry; final analytic sample *N* = 3,462; MASLD *n* = 1,335	LFS; MASLD defined as LFS > − 0.640	38.6% (1,335/3,462)	No significant association between MASLD and FEV1, FVC, or FEV1/FVC; MR also showed no causal association between MASLD and COPD or asthma	Age, sex, BMI, smoking, blood pressure, glucose parameters, lipid profile
Liu et al., 2025 [18]	United Kingdom/UK Biobank (baseline 2006–2010; follow-up to 2023)	Prospective population-based cohort	Adults aged 39–69 years without respiratory disease at baseline; *N* = 393,416	FLI ≥ 60; MASLD defined as steatotic liver disease plus ≥ 1 cardiometabolic risk factor; fibrosis severity by NFS	34.9% (137,295/393,416)	MASLD was independently associated with increased risk of 9/11 respiratory outcomes, including influenza (HR 1.29), pneumonia (HR 1.26), chronic lower respiratory diseases (HR 1.30), asthma (HR 1.22), interstitial lung disease (HR 1.34), pulmonary embolism (HR 1.23), lung and bronchus cancer (HR 1.21), and respiratory system death (HR 1.11); risk increased with greater fibrosis severity	Sex, age, deprivation index, alcohol intake, smoking, BMI, diabetes, hypertension, triglycerides, cholesterol, glucose, ALT, AST, FEV1/FVC, NO₂, NOx, PM2.5, PM10
Sun et al., 2025 [29]	USA/NHANES 2001–2018	Cross-sectional population-based study	Adults ≥ 20 years; asthma defined by self-reported physician diagnosis; *N* = 16,223 (asthma *n* = 2,192)	FLI analyzed in quartiles	Not reported as binary prevalence	A U-shaped association was observed between FLI and asthma risk. Compared with Q3 (FLI 54–83), asthma risk was higher in Q1 OR 1.35 (95% CI 1.01–1.81) and Q4 OR 1.48 (95% CI 1.27–1.73). Lowest asthma risk occurred at FLI ≈ 65 overall (68 in men; 63 in women)	Age, sex, race, education, alcohol, smoking, family history, BMI, diabetes, HDL-cholesterol
Yu et al., 2025 [35]	USA/NHANES 2017–2020 + GWAS consortia	Cross-sectional population-based study + two-sample MR	Adults ≥ 20 years; OSA risk assessed by validated sleep questionnaire; *N* = 6,215	VCTE/FibroScan; CAP ≥ 274 dB/m defined NAFLD	Weighted NAFLD prevalence 43.0%; weighted OSA prevalence 43.1%	OSA was associated with NAFLD in unadjusted and partially adjusted models: OR 1.86 (95% CI 1.63–2.11) and OR 1.69 (95% CI 1.49–1.91), but not after full adjustment: OR 1.07 (95% CI 0.89–1.22). MR suggested a possible causal effect of genetically predicted OSA on NAFLD: IVW OR 1.066 (95% CI 1.010–1.125), although sensitivity analyses attenuated significance	Sex, age, race, education, waist circumference, HbA1c, glucose, total cholesterol, HDL, triglycerides, LDL, marital status, PIR, smoking, BMI, hypertension, diabetes
Tseng et al., 2025 [26]	Taiwan/Taipei Veterans General Hospital	Retrospective cohort study	Adults with obstructive lung disease, including COPD and asthma, who underwent spirometry, impulse oscillometry, and abdominal CT; *N* = 2,572; MASLD *n* = 349	Abdominal CT; hepatic steatosis defined according to 2023 MASLD criteria	13.6% (349/2,572)	MASLD was independently associated with small airway dysfunction (AX > 0.44). Patients with both MASLD and small airway dysfunction had the highest risk of acute exacerbation (34.8%); hepatic steatosis appeared to be the major driver of exacerbation risk	Age > 65 years, smoking, eGFR, NLR, albumin, sex
Associations of Respiratory Diseases with MASLD							
Bocchino et al., 2015 [38]	Italy/Federico II University of Naples	Pilot cross-sectional study	IPF *N* = 29; healthy controls *N* = 15	Conventional liver ultrasound plus transient ultrasound elastography	Mild/moderate steatosis: 27.5% in IPF vs. 6.6% in controls; severe steatosis: 7% in IPF; increased liver stiffness in 38% of IPF patients	Mean liver stiffness was higher in IPF than in controls (6.4 ± 2.2 vs. 5.2 ± 0.4 kPa; *p* = 0.02), suggesting greater hepatic fibrotic burden in IPF	No formal adjustment reported; groups matched for age and sex
Jawa et al., 2021 [32]	Saudi Arabia/King Abdulaziz University Hospital	Retrospective observational study	Adults with confirmed OSA; *N* = 133	Abdominal ultrasonography; ALT; fibrosis risk by NFS and FIB-4	Radiologically suspected NAFLD 44.4%; biochemically suspected NAFLD 63.9%	NAFLD was common among OSA patients, but OSA severity (AHI) was not independently associated with hepatic steatosis or higher fibrosis risk; age and BMI predicted high NFS, whereas cirrhosis predicted high FIB-4	Age, sex, OSA severity, BMI, cirrhosis, elevated ALT, diabetes, dyslipidemia, hypertension
Jördens et al., 2021 [28]	Germany/IQVIA Disease Analyzer database, 2005–2019	Retrospective propensity score–matched cohort	Adults with pulmonary hypertension and matched controls without prior liver disease; total *N* = 18,910 (9,455 PH; 9,455 controls)	NAFLD and cirrhosis identified by ICD-10 codes	10-year cumulative NAFLD incidence 7.3% in PH vs. 3.5% in controls; cirrhosis 1.4% vs. 1.1%	Pulmonary hypertension was independently associated with higher incident NAFLD: HR 1.71 (95% CI 1.39–2.09). The association was stronger in women (HR 1.93) and in patients aged > 80 years (HR 3.30). The increase in cirrhosis incidence was not statistically significant	Propensity score matching based on age, sex, yearly consultation frequency, obesity, diabetes, heart failure, and lipid metabolism disorders
Tsutsumi et al., 2022 [24]	Japan/Health check-up cohort	Cross-sectional observational analysis	Men with ultrasonography-confirmed fatty liver; COPD/high-risk COPD by spirometry; *N* = 2,041, COPD *n* = 420 (20.6%)	Abdominal ultrasonography; MAFLD diagnosed according to international consensus criteria	MAFLD prevalence 90.5% in COPD vs. 85.9% in non-COPD	MAFLD was independently associated with COPD presence: OR 1.46 (95% CI 1.02–2.10; *p* = 0.0385). Decision-tree analysis identified MAFLD as an important classifier for COPD, particularly in non-heavy smokers and men aged < 50 years	Age, smoking exposure
Zheng et al., 2024 [22]	USA/NHANES 2017–2020	Cross-sectional population-based study	Adults ≥ 18 years; COPD *n* = 693, non-COPD *n* = 7,229; total *N* = 7,922	VCTE; hepatic steatosis assessed by CAP (dB/m)	Binary prevalence not reported; mean CAP higher in COPD	Mean CAP was higher in COPD than in non-COPD (277.4 ± 67.3 vs. 264.4 ± 62.2 dB/m). Steatosis was independently associated with COPD prevalence: OR 1.03 per 10 dB/m CAP increase (95% CI 1.01–1.05). A U-shaped association was reported, with the lowest COPD prevalence at CAP 264.85 dB/m	Age, sex, race, BMI, smoking, ALT, ALP, AST, diabetes, PIR, hypertension, asthma, vigorous activity, alcohol
Beibei et al., 2026 [36]	USA/NHANES 2005–2008 + FinnGen	Cross-sectional analysis + bidirectional two-sample MR	NHANES adults *N* = 3,448; FinnGen OSA cases 38,998/controls 336,659; MAFLD cases 2,275/controls 375,002	USFLI; MAFLD defined as ≥ 30	33.9% (1,169/3,448)	OSA was independently associated with MAFLD: adjusted OR 1.96 (95% CI 1.53–2.52). MR supported a causal effect of OSA on MAFLD, with no evidence of reverse causality	Age, sex, race, education, smoking, hypertension, hyperlipidemia, diabetes mellitus
Trzepizur et al., 2026 [34]	Multicenter cohort	Prospective study with polysomnography and liver biopsy	Biopsy-proven NAFLD *N* = 97; advanced fibrosis *n* = 40; moderate-to-severe OSA *n* = 63	Liver biopsy scored using NASH-CRN	Advanced fibrosis 41%; moderate-to-severe OSA 65% overall and 82% among those with F ≥ 3	Moderate-to-severe OSA was independently associated with advanced fibrosis: adjusted OR 3.48 (95% CI 1.03–11.83; *p* = 0.045), but not with simple steatosis	Sex, age, diabetes, obesity
Evidence Suggestive of Bidirectional Associations							
Moon et al., 2018 [23]	South Korea/KNHANES 2007–2010	Cross-sectional population-based analysis	Adults ≥ 20 years; OLD defined as FEV1/FVC < 0.7; *N* = 11,738	LFS; NAFLD defined as LFS > − 0.640	Overall NAFLD prevalence 30.2%; NAFLD more frequent in OLD vs. non-OLD (33.7% vs. 30.0%)	Bidirectional association observed: NAFLD independently associated with OLD OR 1.334 (95% CI 1.108–1.607), and OLD independently associated with NAFLD OR 1.556 (95% CI 1.288–1.879)	Age, sex, smoking
Jullian-Desayes et al., 2021 [31]	France/French university sleep and respiratory centers	Individual participant data meta-analysis	Adults referred for suspected OSA; *N* = 2,120	HSI; hepatic steatosis defined as HSI > 36	75.0% (1,584/2,120)	Steatosis prevalence increased with OSA severity from 51% in no OSA to 85% in severe OSA. OSA severity was independently associated with hepatic steatosis: AHI 5–30 vs. < 5 OR 2.33; AHI > 30 vs. < 5 OR 2.80. COPD stage was not independently associated, although overlap of severe OSA and mild COPD increased steatosis risk	BMI, age, sex, diabetes, hypertension, dyslipidemia
Ding et al., 2023 [37]	FinnGen + European NAFLD GWAS	Two-sample Mendelian randomization	OSA: 16,761 cases/201,194 controls; NAFLD: 1,483 cases/17,781 controls	Genetic instruments; NAFLD defined by GWAS consortium	Not applicable	No robust causal association between genetically predicted OSA and NAFLD after removal of obesity-related SNPs: IVW OR 0.65 (95% CI 0.18–2.37)	Age, sex, genotyping chip, genetic relationship

*AHI* apnea–hypopnea index, *ALP* alkaline phosphatase, *ALT* alanine aminotransferase, *AST* aspartate aminotransferase, *BMI* body mass index, *CAP* controlled attenuation parameter, *CI* confidence interval, *COPD* chronic obstructive pulmonary disease, *FLI* fatty liver index, *HSI* hepatic steatosis index, *HR* hazard ratio, *ILD* interstitial lung disease, *IPF* idiopathic pulmonary fibrosis, *LFS* liver fat score, *MASLD* metabolic dysfunction-associated steatotic liver disease, *MAFLD* metabolic dysfunction-associated fatty liver disease, *MR* Mendelian randomization, *NAFLD* non-alcoholic fatty liver disease, *NASH* non-alcoholic steatohepatitis, *NFS* NAFLD fibrosis score, *OLD* obstructive lung disease, *OR* odds ratio, *OSA* obstructive sleep apnea, *PH* pulmonary hypertension, *PoPH* portopulmonary hypertension, *SAD* small airway dysfunction, *VCTE* vibration-controlled transient elastography

The terms NAFLD, MAFLD, and MASLD are retained as originally used in the included studies

### Epidemiological Evidence: High Prevalence of MASLD in COPD Patients

Clinical cohort data indicate that MASLD is highly prevalent among patients with COPD. Notably, the 2026 Global Initiative for Chronic Obstructive Lung Disease (GOLD) report states that MASLD affects 30–60% of patients with COPD, likely reflecting shared risk factors such as chronic inflammation, obesity, and physical inactivity, as well as potential causal mechanisms including hypoxemia [39].

In a French outpatient COPD cohort (*n* = 111), moderate–to-severe steatosis (≥ S2 by SteatoTest) was identified in 41.4% of patients, with prevalence increasing with GOLD stage: 11.8% in GOLD I, 47.0% in GOLD II, and 46.4% in GOLD III–IV [14]. Steatosis was independently associated with COPD severity. Compared to GOLD I, the odds ratio (OR) for steatosis was 21.80 (95% CI) 2.36–201.02) for GOLD II and 26.77 (95% CI 2.34–306.29) for GOLD III–IV [14].

A subsequent 5-year prospective study in the same cohort confirmed a persistent steatosis prevalence of approximately 41%, although baseline lung function did not significantly differ between individuals with and without steatosis [15]. Notably, liver fibrosis, but not steatosis or NASH, was independently associated with increased cardiovascular events and mortality (HR 2.94, 95% CI 1.18–7.33) during follow-up [15].

Population-level data reinforce this association. In a cross-sectional analysis of NHANES 2017–2020 (*n* = 7,922), steatosis measured by vibration-controlled transient elastography was independently associated with COPD prevalence. Specifically, each 10 dB/m increase in CAP was associated with a 3% increase in COPD odds (OR 1.03, 95% CI 1.01–1.05), with a nonlinear U-shaped relationship also observed [22].

Moreover, in the UK Biobank prospective cohort (*n* = 393,416), MASLD, defined using FLI and cardiometabolic criteria, was independently associated with chronic lower respiratory diseases (HR 1.30), as well as respiratory system death (HR 1.11), during long-term follow-up [18].

Evidence supporting bidirectionality is provided by the Korean KNHANES data (*n* = 11,738), where NAFLD was independently associated with obstructive lung disease (OR 1.334, 95% CI 1.108–1.607), and conversely, obstructive lung disease was associated with NAFLD (OR 1.556, 95% CI 1.288–1.879) [23]. Similarly, in a Japanese health check-up cohort of 2,041 men, MAFLD prevalence was 90.5% among participants with COPD versus 85.9% among those without COPD (*p* = 0.0148), and MAFLD independently predicted COPD (OR 1.46, 95% CI 1.02–2.10) [24].

### Impact on Lung Function: Association Between Liver Fat and Airflow Limitation

The relationship between hepatic steatosis and lung function extends beyond simple comorbidity. A systematic review and meta-analysis of six observational studies (*n* = 133,707) demonstrated that NAFLD is associated with significant reductions in both predicted FEV₁ (pooled weighted mean difference − 2.43%, 95% CI − 3.28 to − 1.58) and predicted FVC (pooled weighted mean difference − 2.96%, 95% CI − 4.75 to − 1.17), suggesting a predominantly restrictive rather than obstructive ventilatory pattern [40].

In a biopsy-proven NAFLD cohort (*n* = 420), fibrosis severity was independently associated with reduced post-bronchodilator FVC expressed as percent predicted. Specifically, the β coefficient of − 4.41 per fibrosis stage (95% CI − 8.39 to − 0.43; *p* = 0.031), after full adjustment for metabolic confounders [24]. Longitudinal data further support this association. Hepatic fibrosis (assessed by FIB-4 score ≥ 1.30) was associated with accelerated FVC decline (− 27.4 vs. − 21.7 mL/year in men; −27.9 vs. − 22.4 mL/year in women; both *p* < 0.02), whereas steatosis alone was not associated with accelerated lung function decline [24].

Although Viglino et al. [14] did not observe significant differences in FEV_1_ or FEV_1_/FVC between COPD patients with and without steatosis at baseline, other datasets suggest subtle functional alterations. In a Taiwanese cohort of 2,572 patients with obstructive lung disease, MASLD, defined by CT, was independently associated with small airway dysfunction (AX > 0.44), a marker of peripheral airway impairment [26]. Importantly, patients with both MASLD and small airway dysfunction exhibited the highest risk of acute exacerbations. Additional evidence from a retrospective study (*n* = 1,391) showed that hepatic steatosis is associated with increased peripheral airway resistance, and that elevated neutrophil-to-lymphocyte ratio combined with decreased lymphocyte-to-monocyte ratio is an independent risk factor for peripheral airway dysfunction in patients with hepatic steatosis [41].

These findings suggest that steatosis, particularly when accompanied by fibrosis or systemic inflammation, may contribute to both restrictive (reduced FVC) and peripheral obstructive changes, potentially through systemic inflammatory signaling and altered metabolic–immune crosstalk.

### Clinical Outcomes: Exacerbations and Cardiovascular–Pulmonary Complications

Steatotic liver disease appears to worsen clinical outcomes in obstructive lung disease. In the Taiwanese cohort, patients with MASLD and small airway dysfunction experienced acute exacerbations in 34.8% of cases, representing the highest-risk clinical phenotype [26]. Hepatic steatosis, rather than fibrosis, was identified as the primary driver of exacerbation risk in this population.

Beyond exacerbations, hepatic fibrosis has been associated with increased cardiovascular events and mortality in COPD patients. In a 5-year prospective study of 111 COPD patients, liver fibrosis, but not steatosis or NASH, was independently associated with cardiovascular events and death (HR 2.94, 95% CI 1.18–7.33), suggesting that advanced liver disease may confer additional prognostic risk beyond hepatic steatosis alone [15].

In advanced liver disease, pulmonary vascular complications become clinically important. In a Turkish single-center study of 171 patients with NASH-related cirrhosis, 25.1% had portopulmonary hypertension (PoPH). Although all patients had advanced steatotic liver disease, PoPH was associated with higher BMI, Model for End-Stage Liver Disease (MELD) scores, bilirubin, triglycerides, and Thyroid Stimulating Hormone (TSH) levels. In multivariable analysis, elevated TSH was the only independent predictor of PoPH (OR 1.18, 95% CI 1.01–1.43) [27].

Additionally, MASLD was associated with an increased risk of pulmonary embolism (HR 1.23) and respiratory mortality (HR 1.11) in the UK Biobank cohort, underscoring the systemic vascular and thrombo-inflammatory burden associated with steatotic liver disease [18]. Importantly, the risk increased with higher liver fibrosis severity, as assessed by the NFS [18].

In a large retrospective propensity score–matched cohort study using the German IQVIA Disease Analyzer database (2005–2019), Jördens et al. [28] evaluated 18,910 adults, including 9,455 patients with pulmonary hypertension and 9,455 matched controls without prior liver disease. NAFLD and liver cirrhosis were identified using ICD-10 diagnostic codes. Over a 10-year follow-up, the cumulative incidence of NAFLD was significantly higher among patients with pulmonary hypertension compared with controls (7.3% vs. 3.5%), whereas cirrhosis incidence was 1.4% versus 1.1%, respectively. Pulmonary hypertension was independently associated with an increased risk of incident NAFLD (HR 1.71, 95% CI 1.39–2.09), with stronger associations observed in women (HR 1.93) and in individuals older than 80 years (HR 3.30) [28]. Although a numerical increase in cirrhosis risk was noted in the pulmonary hypertension group, this association did not reach statistical significance. Overall, these findings suggest a lung-to-liver relationship, wherein pulmonary hypertension may promote hepatic steatosis through mechanisms such as hepatic congestion, systemic inflammation, or shared metabolic dysfunction.

### Asthma: Metabolic Dysfunction and Airway Inflammation

Current evidence supports an association between hepatic steatosis and asthma incidence. In a nationwide Korean cohort (*n* = 160,603), NAFLD defined by FLI was independently associated with adult-onset asthma (HR 1.25 for highest versus lowest FLI; 95% CI 1.15–1.36), with stronger associations observed in women (HR 1.46) [16].

Similarly, in the UK Biobank cohort, MASLD was independently associated with asthma (HR 1.22), and risk increased with worsening liver fibrosis as assessed by the NFS [18].

In NHANES 2001–2018 (*n* = 16,223), a nonlinear U-shaped association between FLI and asthma was observed. Compared to Q3 (FLI 54–83), asthma risk was higher in both Q1 (OR 1.35, 95% CI 1.01–1.81) and Q4 (OR 1.48, 95% CI 1.27–1.73), with restricted cubic spline analysis identifying the lowest asthma risk at a FLI value of approximately 65 overall (68 in men, 63 in women) [29].

The association between MASLD and asthma may be mediated by shared pathophysiological pathways, including systemic low-grade inflammation, insulin resistance, and proinflammatory/anti-inflammatory cytokine imbalance. Importantly, MRS have not demonstrated a causal relationship between MASLD and asthma (IVW *p* = 0.81), suggesting that the observed epidemiological associations may be driven by shared metabolic risk factors rather than a direct causal pathway. Reverse MR analysis also showed no causal relationship (*P* > 0.05) [30].

The association between NAFLD and asthma incidence was also more pronounced in women (HR 1.46; 95% CI 1.13–1.64) than in men (HR 1.07; 95% CI 0.94–1.20), suggesting potential sex-specific metabolic–inflammatory interactions that warrant further investigation [16].

In summary, the risk of asthma increases with the severity of MASLD as assessed by the NFS, suggesting a dose-response relationship between liver disease severity and respiratory outcomes. In the UK Biobank cohort, this relationship was independent of polygenic risk scores and related risk alleles [18].

### OSA: Chronic Intermittent Hypoxia and MASLD Progression

The MASLD–OSA relationship is among the most robust and biologically plausible associations identified in the current literature. In a Korean nationwide cohort of over 8 million individuals, NAFLD independently increased the risk of incident OSA in a dose-dependent manner (adjusted hazard ratio (aHR) 1.15 for FLI 30–60 and aHR 1.21 for FLI ≥ 60 versus FLI < 30) [17]. Importantly, the association remained significant regardless of BMI and abdominal obesity status, suggesting that NAFLD may contribute to OSA risk independently of central adiposity.

Conversely, in an individual participant data meta-analysis of 2,120 adults referred for suspected OSA, steatosis ((HIS) > 36) was present in 75% of participants, increasing from 51% in individuals without OSA to 85% in those with severe OSA. OSA severity independently predicted hepatic steatosis (OR 2.80 for AHI > 30 vs. < 5) [31]. Additional risk factors for steatosis in this population included BMI > 26 kg/m², older age, type 2 diabetes, and male sex, highlighting the clustering of metabolic risk factors in patients with OSA. In a Saudi cohort of 133 patients with polysomnography-confirmed OSA, radiologic NAFLD was present in 44.4% and elevated ALT were observed in 63.9%. However, OSA severity, based on AHI, was not independently associated with steatosis or high fibrosis risk. Instead, age and BMI predicted higher NFS, while liver cirrhosis predicted elevated FIB-4, emphasizing the dominant role of metabolic factors in fibrosis risk stratification [32].

Biopsy-based analyses have provided further mechanistic insight. In individuals with severe obesity undergoing bariatric surgery, OSA severity and nocturnal hypoxemia were associated with greater lobular inflammation and hepatic fibrosis, particularly among patients without overt metabolic syndrome. Notably, Trzepizur et al. reported no association between OSA and steatosis or steatohepatitis alone [34], suggesting that OSA may preferentially promote fibrosis progression rather than the initial development of steatosis. In this study, moderate-to-severe OSA (AHI ≥ 15) was independently associated with advanced fibrosis (F ≥ 3) with OR 3.48 (95% CI 1.03–11.83; *p* = 0.045) in biopsy-proven NAFLD patients. Similar associations were observed with nocturnal hypoxia markers, including oxygen desaturation index and time spent below 90% oxygen saturation [34].

Mechanistically, intermittent hypoxia, which is a critical consequence of OSA, has been linked to mitochondrial dysfunction, dysregulation of glucose and lipid metabolism, worse insulin resistance, and increased hepatic de novo lipogenesis [42]. Animal models have shown that chronic intermittent hypoxia induces liver fibrosis via TLR4/MyD88/MAPK/NF-κB signaling pathways, even under relatively mild hypoxic conditions [43, 44].

MRS suggest a potential causal contribution of genetically predicted OSA to NAFLD development. However, the available MR evidence remains inconsistent. In a recent analysis combining NHANES 2017–2020 data with two-sample MR, OSA was associated with NAFLD in unadjusted models but lost significance after full metabolic adjustment, although genetically predicted OSA showed a modest potential causal effect (IVW OR 1.066, 95% CI 1.010–1.125], which was attenuated in sensitivity analyses [35]. Similarly, Beibei et al. [36] reported that OSA increased MAFLD risk (aOR 1.96, 95% CI 1.53–2.52 in cross-sectional analysis; IVW OR 1.50, 95% CI 1.07–2.11 in MR study) whereas Zhang et al. found no direct causal association after BMI adjustment [45]. Another MR study found that waist circumference may mediate the connection between OSA and NAFLD [46]. Overall, the inconsistency across MRS suggests that the observed epidemiological association between OSA and MASLD/MAFLD/NAFLD may be driven in part by shared metabolic risk factors, particularly obesity and insulin resistance, rather than by a purely direct causal pathway.

### Interstitial Lung Disease: Shared Fibrotic Pathways

MASLD was independently associated with incident interstitial lung disease (HR 1.34, 95% CI 1.20–1.49) in the UK Biobank study [18]. Importantly, the association was more significant with higher NFS, suggesting that hepatic fibrogenesis may parallel pulmonary fibrotic risk.

A pilot study by Bocchino et al. showed that mean liver stiffness, measured by transient ultrasound elastography, was significantly higher in patients with IPF (*n* = 29) compared to healthy controls (*n* = 15) (6.4 ± 2.2 kPa vs. 5.2 ± 0.4 kPa; *p* = 0.02). Increased liver stiffness, suggestive of hepatic fibrosis, was present in 38% of IPF patients. Additionally, mild-to-moderate hepatic steatosis was identified in 27.5% of patients with IPF compared with 6.6% of controls, while severe steatosis was present in 7% of patients with IPF, supporting the possibility of a relationship between hepatic and pulmonary fibrosis [38]. Although the groups were matched for age and sex, formal multivariable adjustment was not performed, which limits causal interpretation.

## Synthesis of Evidence, Clinical Implications and Future Perspectives

### Synthesis of Findings and Pathophysiological Interpretation

The present systematic review synthesizes the current evidence on the association between MASLD and a broad spectrum of respiratory disorders. Overall, the available literature suggests that MASLD may be linked to several pulmonary conditions, including COPD, asthma, OSA, interstitial lung disease, pulmonary hypertension, and respiratory mortality [14–18, 22–38]. Notably, although bronchiectasis was included among the predefined outcomes of interest, no eligible studies specifically addressing this condition were identified, highlighting an important gap in the current literature.

The strength and consistency of the evidence vary substantially across respiratory phenotypes. While the association appears more consistently supported for some conditions, particularly COPD and OSA, findings for other disorders remain more heterogeneous and less conclusive [14–18, 22–38]. Importantly, the interpretation of these associations should be considered in the context of study quality. The most consistent and robust findings, particularly for COPD, OSA, and composite respiratory outcomes, are primarily supported by large population-based cohort studies and analyses derived from nationwide datasets, many of which were assessed as having low to moderate risk of bias. These studies typically incorporated extensive multivariable adjustment and large sample sizes, enhancing the reliability of their findings. In contrast, evidence derived from smaller clinical cohorts or highly selected populations, including sleep clinic or bariatric surgery cohorts, as well as cross-sectional analyses, should be interpreted more cautiously due to moderate risk of bias, potential selection bias, and the absence of temporal inference. Notably, no cross-sectional study was classified as unequivocally low risk of bias, reflecting inherent limitations of this design. MR studies, although methodologically rigorous, yielded inconsistent results across respiratory outcomes, further highlighting the complexity of the observed associations.

The interpretation of MR findings in this context requires particular caution. Although MR offers an approach to strengthen causal inference by leveraging genetic instruments, its validity depends on key assumptions, including the absence of horizontal pleiotropy and the appropriate selection of instrumental variables. In the included studies, MR results were not consistent across respiratory outcomes and appeared sensitive to analytical choices, such as adjustment for obesity-related traits and the handling of pleiotropic variants. In several analyses, initially significant associations were attenuated after sensitivity analyses or exclusion of outlier instruments, suggesting that shared genetic determinants, particularly those related to adiposity and metabolic dysfunction, may partly account for the observed relationships. Furthermore, variation in phenotype definitions (e.g. questionnaire-based versus polysomnography-defined OSA) may introduce additional heterogeneity. Therefore, MR findings should be interpreted as supportive but not definitive evidence of causality, and should be considered alongside observational and mechanistic data.

An additional important source of heterogeneity relates to the use of different diagnostic frameworks for steatotic liver disease, including NAFLD, MAFLD, and MASLD. Although these terms are often used interchangeably in the literature, they are not fully equivalent. NAFLD is defined by the exclusion of secondary causes of hepatic steatosis, whereas MAFLD and MASLD adopt a positive definition based on the presence of metabolic dysfunction. In particular, MAFLD criteria may preferentially identify individuals with a higher cardiometabolic burden, potentially enriching study populations for systemic inflammation and metabolic comorbidity. As a result, studies using MAFLD definitions may report stronger associations with respiratory outcomes compared to those using NAFLD criteria. Conversely, NAFLD-based definitions may include more heterogeneous populations, including individuals without overt metabolic dysfunction. These differences may contribute to variability in effect estimates, limit comparability across studies, and introduce potential misclassification when synthesizing evidence. Importantly, in the present review, all diagnostic terms were retained as originally defined in the included studies, and findings were interpreted within the context of these evolving definitions.

Although heterogeneity across study designs and diagnostic definitions precluded quantitative synthesis, several important patterns emerge. First, MASLD appears highly prevalent among patients with COPD and OSA, and its presence is associated with more severe respiratory phenotypes in several cohorts [14, 15, 17, 31]. While shared risk factors such as obesity and smoking contribute to this overlap, most large cohort studies adjusted for major metabolic and lifestyle confounders, including BMI, smoking status, age, sex, diabetes, hypertension, dyslipidemia, alcohol intake, and, in some datasets, markers of insulin resistance and systemic inflammation. Notably, the associations between MASLD and respiratory outcomes persisted after multivariable adjustment, suggesting that qualitative adipose tissue dysfunction, often referred to as “meta-inflammation”, and hepatokine-mediated signaling may exert effects beyond simple somatometric indices of obesity. Importantly, several population-based studies demonstrate that this relationship remains significant after adjustment for traditional metabolic confounders [18, 22–24], supporting the concept that hepatic steatosis may reflect an active component of systemic metabolic dysfunction rather than a passive epiphenomenon.

Second, the relationship between liver disease and lung function extends beyond simple comorbidity. Evidence suggests that hepatic fibrosis, in particular, may be linked to functional impairment, including reduced lung volumes and peripheral airway dysfunction [25, 26]. These findings support the hypothesis that progressive liver injury, rather than isolated steatosis, may contribute to systemic inflammatory and fibrotic signaling that impacts pulmonary structure and function. Advanced fibrosis may represent a cumulative index of chronic metabolic stress, integrating adipose tissue–derived inflammatory signaling, oxidative injury, and sustained activation of profibrotic pathways, notably TGF-β signaling [47, 48].

In addition to classical cytokine-mediated inflammation, emerging data support a role for hepatokine-mediated interorgan communication within the liver–lung axis. Hepatokines, such as fibroblast growth factor 21 (FGF-21), fetuin-A, and angiopoietin-like proteins are dysregulated in MASLD and may modulate systemic insulin sensitivity, endothelial function, and inflammatory tone [9, 11]. Although FGF-21 is initially considered a compensatory metabolic hormone, persistent elevation may reflect metabolic stress and has been linked with fibrotic remodeling and oxidative pathways [9–11]. Moreover, platelet-derived growth factor (PDGF) signaling, a central driver of hepatic stellate cell activation and fibrogenesis, shares profibrotic downstream cascades with pulmonary fibroblast activation, including TGF-β–mediated extracellular matrix deposition. These shared hepatokine and growth factor pathways provide a plausible mechanistic substrate for parallel hepatic and pulmonary fibrotic remodeling beyond simple obesity-related inflammation [9, 11]. In asthma, associations appear more closely related to shared metabolic and inflammatory dysregulation rather than direct hepatic injury per se [16, 18, 28]. Moreover, in obesity-associated asthma phenotypes, leptin-mediated airway hyperresponsiveness, reduced adiponectin signaling, and systemic IL-6 activation may amplify airway inflammation, providing a plausible biological bridge between hepatic steatosis and asthma risk [49].

Third, the MASLD–OSA relationship is among the most robust and biologically plausible associations identified [17, 31–37]. Chronic intermittent hypoxia, a hallmark of OSA, is known to promote hepatic lipid accumulation, oxidative stress, and fibrogenesis. Conversely, obesity, systemic inflammation and metabolic dysfunction in MASLD may predispose to upper airway collapsibility and OSA development. Nevertheless, MRS have yielded inconsistent results, particularly after adjustment for BMI, suggesting that chronic low-grade inflammation and insulin resistance remain major drivers of the observed epidemiological overlap [35–37]. This inconsistency likely reflects the complex interplay between obesity, upper airway mechanics, intermittent hypoxia, and hepatic lipid metabolism, rather than a simple unidirectional causal pathway.

The association between MASLD and interstitial lung disease, including IPF, is an emerging area of research interest [15, 35]. Converging mechanistic data indicate shared profibrotic pathways involving TGF-β signaling, oxidative stress, lipid metabolic reprogramming, and extracellular matrix remodeling [50–54]. Moreover, obesity-related mitochondrial dysfunction, altered fatty acid oxidation, and extracellular matrix remodeling may further contribute to a systemic profibrotic milieu affecting both hepatic and pulmonary parenchyma [55]. These parallels support the concept of a systemic fibrotic predisposition in metabolically dysregulated states, although clinical evidence remains limited and requires prospective validation.

At the advanced disease spectrum, pulmonary vascular complications further illustrate the clinical relevance of the liver–lung axis. PoPH represents a severe complication of advanced liver disease, and emerging data suggest that even early pulmonary hemodynamic alterations may influence prognosis. These findings underscore the systemic vascular and thrombo-inflammatory burden associated with metabolic and fibrotic liver disease [27, 28].

Overall, the findings may support a metabolically mediated liver–lung axis. MASLD should therefore be considered not only a metabolic comorbidity but potentially a modifier of respiratory disease risk and phenotype. Within this model, MASLD may function less as an isolated hepatic disease and more as a measurable manifestation of systemic adipose tissue dysfunction, reflecting the intensity of metabolic stress imposed on multiple organ systems.

From a clinical perspective, weight reduction represents a shared therapeutic target across several conditions. The Global Initiative for Asthma (GINA) recommends that weight reduction should be included in the treatment plan for patients with obesity and asthma (Evidence level B), noting that even 5–10% weight loss may lead to improved asthma control and quality of life, with the most striking results observed after bariatric surgery [56].

**Fig. 2:**
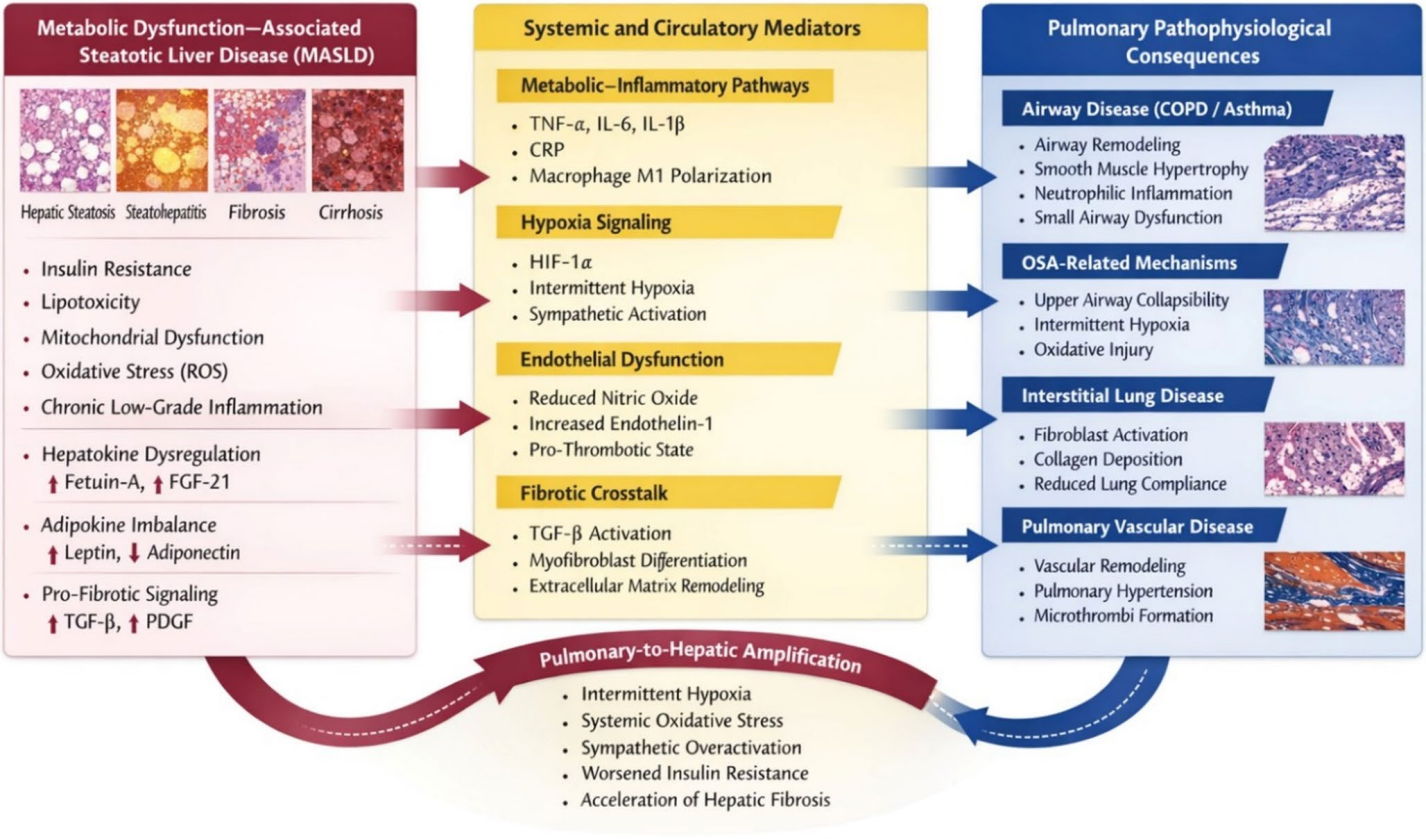
The liver–lung axis in MASLD. MASLD promotes systemic metabolic and inflammatory dysregulation characterized by insulin resistance, oxidative stress, hepatokine imbalance, and pro-fibrotic signaling. Circulating inflammatory cytokines, hypoxia-inducible pathways, endothelial dysfunction, and extracellular matrix remodeling contribute to airway inflammation, small airway dysfunction, interstitial fibrosis, and pulmonary vascular remodeling. Conversely, pulmonary disorders, particularly obstructive sleep apnea, induce intermittent hypoxia and systemic oxidative stress that may accelerate hepatic steatosis progression and fibrogenesis

Evidence further indicates that metabolic improvement through lifestyle or bariatric interventions may favorably influence asthma control [57], reinforcing the interconnected nature of hepatic and pulmonary metabolic dysregulation. Figure 2 summarizes the liver–lung axis in MASLD.

**Fig. 3:**
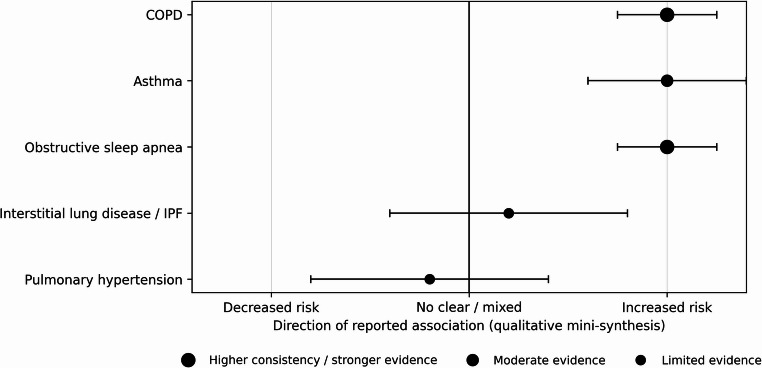
Qualitative forest-style summary of associations between MASLD and major respiratory disorders. Interpretation: Points to the right of the null line indicate that the majority of included studies report an increased risk/prevalence of the respiratory disorder among individuals with MASLD; points near the null line indicate mixed or inconclusive results. Larger points denote higher consistency and stronger supporting evidence (based on number/size of adjusted cohort studies and concordance of direction), while smaller points indicate limited evidence. This visualization is intended as an overview of the evidence and should not be interpreted as a pooled effect estimate

To provide an at-a-glance synthesis across respiratory phenotypes, Fig. 3 presents a qualitative forest-style summary of the reported associations between MASLD and each major respiratory disorder. This is not a formal meta-analysis; however, it summarizes the direction of association and the relative consistency of findings across the included adjusted human studies in the presence of substantial clinical and methodological heterogeneity.

Importantly, while many associations are consistent and biologically plausible, causality remains uncertain. MRS have not consistently confirmed direct causal effects for COPD or asthma [25, 30–32], emphasizing that shared cardiometabolic risk factors may account for much of the observed overlap. Thus, the liver–lung axis may represent a network of interconnected pathophysiological mechanisms and not a strictly linear causal pathway.

### Implications for Clinical Practice

Available evidence does not yet mandate universal respiratory screening for all patients with MASLD; however, it supports a low threshold for targeted case-finding in hepatology clinics, particularly when fibrosis is advanced or cardiometabolic comorbidities cluster. In practical terms, integrating brief symptom-based tools and structured referral pathways may help identify clinically actionable respiratory disease without promoting unnecessary overtesting [15, 17, 23].

Given the strong association between MASLD and OSA, as well as the link between OSA-related intermittent hypoxia and liver fibrosis progression, opportunistic screening may be considered in selected MASLD patients with obesity, type 2 diabetes mellitus, resistant hypertension, atrial fibrillation, excessive daytime sleepiness, loud habitual snoring, or witnessed apneic episodes during sleep. The American Association for the Study of Liver Diseases (AASLD) Practice Guidance suggests that screening for OSA may be considered in patients with MASLD and overweight or obesity, particularly in the presence of clinical risk factors [58]. A short, validated questionnaire (for example, the STOP-Bang questionnaire or the Berlin Questionnaire) combined with the Epworth Sleepiness Scale may effectively triage patients for formal sleep evaluation, such as overnight polysomnography or home sleep apnea testing. Particular attention should be paid to patients with suspected or established MASH and those with elevated noninvasive fibrosis scores.

The stage of liver fibrosis may serve as a useful trigger for pulmonary evaluation, as fibrosis appears to be more strongly associated with pulmonary functional impairment than hepatic steatosis alone [25, 30, 40]. Therefore, patients with advanced fibrosis (stage ≥F3) or cirrhosis may benefit from structured respiratory assessment [25, 58]. This evaluation should focus on respiratory symptoms, including exertional dyspnea and reduced exercise tolerance, measurement of resting and exertional oxygen saturation by pulse oximetry, and baseline spirometry when clinically indicated. A detailed smoking history and assessment of occupational or environmental inhalational exposures are essential components of this evaluation [39, 58].

In patients with cirrhosis, clinicians should additionally consider liver-specific pulmonary syndromes, including hepatopulmonary syndrome and PoPH, and employ transthoracic echocardiography and arterial blood gas analysis where clinically indicated. Screening of liver transplant candidates by pulse oximetry is indicated to detect hepatopulmonary syndrome, using a threshold value of SpO₂ <96% at sea level to trigger further evaluation [59]. Current practice guidelines also recommend obtaining an echocardiogram in all patients with portal hypertension to assess for pulmonary hypertension, and the American Society of Transplantation recommends screening for PoPH with echocardiography as part of a comprehensive pre–liver transplant evaluation [59–61].

For individuals with non-cirrhotic advanced fibrosis, a useful approach is to prioritize pulmonary function testing in those who report respiratory symptoms or have known COPD or asthma. At the same time, clinicians should remain vigilant for restrictive ventilatory physiology or unexplained hypoxemia which may suggest an interstitial lung disease phenotype.

The overarching screening strategy parallels that used in cirrhosis of other etiologies. Doppler transthoracic echocardiography remains the preferred first-line noninvasive test when pulmonary hypertension is suspected and is routinely recommended in the evaluation of liver transplant candidates to detect PoPH [59, 60]. What may differ in the context of cirrhosis due to MASH or MASLD is the higher pre-test probability of cardiopulmonary comorbidities and the presence of competing contributors to dyspnea, such as heart failure with preserved ejection fraction (HFpEF), obesity hypoventilation syndrome, and concomitant OSA [7, 17, 27, 31, 58, 62]. These conditions may mimic or exacerbate pulmonary vascular disease.

Accordingly, a lower threshold for comprehensive cardiopulmonary evaluation, including transthoracic echocardiography and measurement of circulating natriuretic peptides when appropriate, and multidisciplinary interpretation is warranted [7, 27, 58, 59], particularly in patients presenting with disproportionate dyspnea, reduced diffusing capacity of the lung for carbon monoxide (DLCO), oxygen desaturation, or clinical signs suggestive of right ventricular strain [27, 39, 59]. Recent evidence indicates that even early-stage PoPH, defined by the 2022 ESC/ERS criteria (mPAP 20.5–24.5 mmHg with pulmonary vascular resistance > 2 Wood units), is associated with markedly increased mortality in patients with cirrhosis, highlighting the importance of early recognition and targeted intervention [63].

However, it should be emphasized that the majority of the available evidence is derived from observational studies, and therefore, these considerations should be interpreted as supportive of clinical awareness rather than definitive recommendations for routine screening or management.

### Strengths

This review has several strengths. First, to the best of our knowledge, it represents the first systematic synthesis specifically addressing the association between MASLD and the full spectrum of major respiratory diseases in adults. Second, the review incorporates diverse study designs, including large nationwide cohorts and biopsy-based analyses, allowing for evaluation across different disease severities and populations. Third, careful attention was paid to diagnostic definitions of both liver and respiratory diseases, allowing meaningful comparison and characterization of methodological heterogeneity. Fourth, the use of validated risk-of-bias tools (the NOS for cohort studies and the Joanna Briggs Institute Critical Appraisal Checklist for cross-sectional studies) further enhances the methodological rigor of the synthesis. Finally, the review places epidemiological findings within a broader mechanistic model, integrating emerging insights into metabolic inflammation, hypoxia signaling, and shared fibrotic pathways.

### Limitations

Several limitations warrant consideration, particularly the observational nature of most included studies and substantial heterogeneity across study designs. First, definitions of MASLD/MAFLD/NAFLD varied considerably, ranging from imaging-based diagnosis to non-invasive indices and administrative coding. Similarly, respiratory outcomes were defined using different criteria, including spirometry, physician diagnosis, questionnaires, or diagnostic codes. Second, because most studies were observational, causal inference remains limited. Third, fibrosis severity was inconsistently assessed and reported across studies. Given emerging evidence suggesting that liver fibrosis may be more strongly associated with adverse respiratory outcomes than steatosis alone, incomplete staging may have attenuated or obscured clinically relevant associations. Fourth, reverse causality cannot be excluded, particularly in cross-sectional analyses examining relationships between COPD, OSA, and MASLD. Chronic respiratory disease and OSA may promote weight gain, systemic inflammation and hepatic fibrogenesis, while advanced liver disease may reduce activity levels and lung volumes; without longitudinal assessment of both organs, temporality cannot be firmly established.

Fifth, residual confounding is particularly relevant in this literature because obesity, insulin resistance, smoking and physical inactivity are strongly associated with both hepatic and respiratory endpoints. Many datasets lacked granular data on cumulative smoking exposure, alcohol intake, occupational/environmental exposures, physical activity, diet quality, socioeconomic status, and medication use (e.g. GLP-1 receptor agonists (GLP-1RAs), Sodium-Glucose Cotransporter-2 inhibitors (SGLT-2is), and statins), all of which could bias effect estimates even after multivariable adjustment.

Sixth, heterogeneous exposure definitions further limit comparability across studies. Investigations variably used NAFLD, MAFLD, or MASLD definitions, imaging modalities, elastography, serum indices, or administrative coding. In many cohorts, it was not possible to distinguish isolated steatosis from steatohepatitis or to quantify fibrosis severity using standardized methods. Misclassification of both liver disease severity and respiratory phenotypes (particularly when based on diagnostic codes or self-report) likely attenuated true associations and may partly explain discordant findings in MR studies.

Seventh the current evidence base remains predominantly observational, with inherent risks of selection bias, surveillance (detection) bias, and incomplete adjustment for metabolic comorbidity. Prospective cohort studies and interventional trials with standardized fibrosis staging and harmonized pulmonary outcomes are needed before firm screening recommendations can be universally endorsed.

Finally, an additional limitation relates to the use of vote counting based on the direction of effect as part of the qualitative synthesis. Although this approach is recommended when quantitative pooling is not feasible, it does not incorporate the magnitude of effect estimates, confidence intervals, or study precision, and therefore may oversimplify the overall body of evidence. In particular, vote counting assigns equal weight to studies regardless of sample size, methodological quality, or statistical power, which may obscure important differences between large population-based cohorts and smaller or higher-risk-of-bias studies. Furthermore, this method does not allow formal assessment of between-study heterogeneity or publication bias. As a result, the qualitative summary presented in this review should be interpreted with caution, and the direction and consistency of associations should not be equated with the strength or certainty of evidence.

### Future Directions

Future research should prioritize well-designed prospective cohort studies with standardized definitions of MASLD under the updated nomenclature and harmonized respiratory outcome measures [2, 3, 18, 30]. Longitudinal assessment of liver fibrosis progression would provide critical insight into temporality and causality [22, 25, 30]. Mechanistic studies are needed to further elucidate shared metabolic–fibrotic signaling pathways, particularly focusing on hypoxia-inducible factors, adipokine signaling, macrophage metabolic reprogramming, and extracellular matrix remodeling. Multi-omics approaches integrating lipidomics, transcriptomics, and inflammatory profiling may help identify shared molecular signatures across organs [47, 53–55]. Randomized interventional studies targeting metabolic dysfunction, such as weight reduction, GLP-1RAs, SGLT-2 inhibitors, or antifibrotic agents, should incorporate respiratory endpoints to determine whether modification of hepatic disease translates into pulmonary benefit [8, 57, 64–66]. In addition, future research should evaluate the cost-effectiveness of structured respiratory screening strategies in patients with advanced MASLD [18, 25, 58]. Furthermore, clinical guidelines may consider more structured screening strategies, particularly in high-risk populations (e.g. patients with OSA, advanced fibrosis, or pulmonary hypertension), pending further confirmatory evidence.

From a translational perspective, weight reduction and metabolic modulation represent shared therapeutic targets across hepatic and pulmonary disease. Bariatric surgery improves MASLD histology and reduces OSA severity, while GLP-1RAs and emerging dual incretin therapies reduce liver fat content and systemic inflammation, with potential downstream benefits on respiratory outcomes [64]. Resmetirom, a thyroid hormone receptor beta-selective agonist, is the first FDA-approved pharmacotherapy for noncirrhotic MASH with moderate-to-advanced fibrosis (≥ F2), demonstrating significant improvements in both MASH resolution and fibrosis regression in the MAESTRO-NASH trial [65, 66]. Whether such liver-directed therapies confer respiratory benefits remains to be determined and represents an important area for future investigation. Prospective trials incorporating dual hepatic and pulmonary endpoints are needed to determine whether targeting obesity-driven metabolic pathways can attenuate both organ-specific and systemic complications [67, 68].

## Conclusions

The current body of evidence suggests an association between MASLD and several respiratory phenotypes, including COPD, OSA, asthma, interstitial lung disease, pulmonary hypertension, and respiratory mortality. However, the strength, consistency, and direction of these associations differ across conditions, with some respiratory outcomes supported by more robust and concordant evidence than others.

The relationship between MASLD and OSA appears to be the most robust and biologically plausible. MASLD is associated with increased incident OSA risk, whereas OSA-related chronic intermittent hypoxia may promote hepatic steatosis progression and fibrogenesis. In COPD, the association is consistently observed in population-based cohorts, although shared metabolic and smoking-related risk factors likely play major roles. In asthma, associations appear largely epidemiological and seem to be driven primarily by shared metabolic and inflammatory dysregulation rather than a direct causal hepatic effect. In interstitial lung disease, particularly fibrotic phenotypes, the relationship appears mechanistically plausible, potentially reflecting parallel profibrotic signaling pathways in the liver and lung. Pulmonary hypertension represents a more complex scenario in which advanced liver disease may predispose to pulmonary vascular remodeling, while pulmonary hypertension itself may increase the future risk of MASLD.

Importantly, liver fibrosis severity consistently shows stronger associations with impaired lung function and adverse respiratory outcomes than steatosis alone, supporting the concept that MASLD-related fibrogenesis reflects cumulative systemic metabolic stress affecting multiple organs.

Although MRS have not uniformly confirmed direct causality, with analyses showing no causal association between MASLD and pulmonary function, COPD, or asthma (all *P* > 0.05), the convergence of epidemiological, mechanistic, and clinical data may support the existence of a metabolically mediated liver–lung axis. Notably, the relationship between MASLD and respiratory diseases appears independent of polygenic risk scores and related risk alleles, suggesting that the association is not solely driven by shared genetic susceptibility [18]. Within this context, MASLD should be viewed not merely as an isolated hepatic condition but as a marker of systemic adipose tissue dysfunction and metabolic toxicity that may influence respiratory disease risk, phenotype, and prognosis.

Future longitudinal and interventional studies incorporating standardized fibrosis staging and respiratory endpoints will be essential to clarify temporality and determine whether targeting metabolic inflammation and fibrotic signaling may improve outcomes across both hepatic and pulmonary domains.

## Key Annotated References

- Liu B, Jia Y, Gu Z, Li Y, Zhou Y, Cao Y. Metabolic dysfunction associated steatotic liver disease is associated with an increased risk of multiple respiratory system diseases. *Sci Rep.* 2025;15(1):15,937. https://doi.org/10.1038/s41598-025-96710-3. *(of outstanding importance).*
  This large prospective analysis (UK Biobank) showed that MASLD is independently associated with incident respiratory outcomes, including chronic lower respiratory diseases, asthma and interstitial lung disease, with stronger associations across increasing NAFLD Fibrosis Score strata, supporting the relevance of hepatic fibrogenesis to pulmonary risk.
- Chung GE, Cho EJ, Yoo JJ, Chang Y, Cho Y, Park SH, Shin DW, Han K, Yu SJ. Nonalcoholic fatty liver disease is associated with the development of obstructive sleep apnea. *Sci Rep.* 2021;11(1):13473. https://doi.org/10.1038/s41598-021-92703-0. *(of outstanding importance).*
  In a large nationwide cohort, NAFLD (by fatty liver index) predicted incident OSA in a dose-dependent manner, highlighting a clinically strong liver→sleep-disordered breathing link within the obesity-driven liver–lung axis.
- Jullian-Desayes I, Trzepizur W, Boursier J, Joyeux-Faure M, Bailly S, Benmerad M, et al. Obstructive sleep apnea, chronic obstructive pulmonary disease and NAFLD: an individual participant data meta-analysis. *Sleep Med.* 2021;77:357–364.https://doi.org/10.1016/j.sleep.2020.04.004. *(of outstanding importance).*
  This individual participant data meta-analysis showed a high burden of steatosis among patients evaluated for OSA and clarified the clustering and overlap of OSA, COPD and NAFLD/steatosis in obesity-related populations, supporting metabolic–hypoxic crosstalk.
- Lee HW, Lee DH, Lee JK, Lee S, Koo BK, Joo SK, Heo EY, Jung YJ, Kim W, Kim DK. Pulmonary function is associated with fibrosis severity in patients with biopsy-proven nonalcoholic fatty liver disease. *Liver Int.* 2020;40(12):3008–3017. https://doi.org/10.1111/liv.14626. *(of importance).*
  In biopsy-proven NAFLD, fibrosis stage, not steatosis alone, was independently associated with worse pulmonary function, supporting the concept that hepatic fibrogenesis captures cumulative systemic metabolic injury relevant to the lung.
- Tseng CH, Jou BY, Shen HC, Yeh HY, Hong SY, Lin YH, Tsai HC, Li TH, Su CW, Chou KT, Perng DW, Yang YY, Hou MC. Increased risk of acute exacerbation in obstructive airway disease: the impact of metabolic dysfunction-associated steatotic liver disease and small airway dysfunction. *BMJ Open Respir Res.* 2025;12(1):e003352. https://doi.org/10.1136/bmjresp-2025-003352. *(of importance).*
  This cohort study linked MASLD to small airway dysfunction and showed that the MASLD+small airway dysfunction phenotype carried the highest exacerbation risk, suggesting MASLD may modify obstructive airway disease severity beyond shared risk factors.

## Supplementary Information

Below is the link to the electronic supplementary material.

Supplementary Material 1

Supplementary Material 2

Supplementary Material 3

## Data Availability

Data are contained within the article.

## Abbreviations

AHI: Apnea–Hypopnea Index
ALT: Alanine Aminotransferase
AST: Aspartate Aminotransferase
AX: Area of Reactance
BMI: Body Mass Index
CAP: Controlled Attenuation Parameter
CI: Confidence Interval
COPD: Chronic Obstructive Pulmonary Disease
CRP: C-Reactive Protein
CT: Computed Tomography
DLCO: Diffusing Capacity of the Lung for Carbon Monoxide
FEV1: Forced Expiratory Volume in 1 s
FIB-4: Fibrosis-4 Index
FLI: Fatty Liver Index
FVC: Forced Vital Capacity
GOLD: Global Initiative for Chronic Obstructive Lung Disease
GWAS: Genome-Wide Association Study
HIF-1α: Hypoxia-Inducible Factor 1 Alpha
HR: Hazard Ratio
HSI: Hepatic Steatosis Index
ICD-10: International Classification of Diseases, 10th Revision
IL-6: Interleukin-6
IPF: Idiopathic Pulmonary Fibrosis
MAFLD: Metabolic Dysfunction–Associated Fatty Liver Disease
MASLD: Metabolic Dysfunction–Associated Steatotic Liver Disease
MELD: Model for End-Stage Liver Disease
MR: Mendelian Randomization
NAFLD: Nonalcoholic Fatty Liver Disease
NASH: Nonalcoholic Steatohepatitis
NFS: NAFLD Fibrosis Score
NHANES: National Health and Nutrition Examination Survey
OSA: Obstructive Sleep Apnea
OR: Odds Ratio
PE: Pulmonary Embolism
PoPH: Portopulmonary Hypertension
SAD: Small Airway Dysfunction
SGLT2: Sodium-Glucose Cotransporter-2
TGF-β: Transforming Growth Factor Beta
TNF-α: Tumor Necrosis Factor Alpha
TSH: Thyroid-Stimulating Hormone
VCTE: Vibration-Controlled Transient Elastography
