# Supplementary material for: Metabolic Dysfunction–Associated Steatotic Liver Disease and Respiratory Disorders: A Systematic Review of Clinical and Pathophysiological Associations
Source: Curr Obes Rep. 2026 Apr 17;15(1):36. doi: 10.1007/s13679-026-00713-8 (PMC13090296; doi:10.1007/s13679-026-00713-8)
Supplement: Supplementary file 1 — Supplementary Material 1 [file 13679_2026_713_MOESM1_ESM.docx]

**Supplementary Table S1. Full electronic search strategy for PubMed (MEDLINE)**

#1 "Non-alcoholic Fatty Liver Disease"[Mesh]

OR "NAFLD"

OR "non-alcoholic fatty liver disease"

OR "MASLD"

OR "metabolic dysfunction-associated steatotic liver disease"

OR "MAFLD"

OR "metabolic-associated fatty liver disease"

OR "fatty liver"

OR "steatotic liver disease"

OR "hepatic steatosis"

OR "steatohepatitis"

OR "NASH"

#2 "Pulmonary Disease, Chronic Obstructive"[Mesh]

OR "COPD"

OR "chronic obstructive pulmonary disease"

OR "airflow obstruction"

#3 "Asthma"[Mesh]

OR "asthma"

#4 "Sleep Apnea, Obstructive"[Mesh]

OR "obstructive sleep apnea"

OR "OSA"

OR "sleep-disordered breathing"

OR "sleep apnea syndrome"

#5 "Interstitial Lung Diseases"[Mesh]

OR "interstitial lung disease"

OR "pulmonary fibrosis"

OR "idiopathic pulmonary fibrosis"

#6 "Hypertension, Pulmonary"[Mesh]

OR "pulmonary hypertension"

#7 "Pulmonary Embolism"[Mesh]

OR "pulmonary embolism"

#8 "Bronchiectasis"[Mesh]

OR "bronchiectasis"

#9 #2 OR #3 OR #4 OR #5 OR #6 OR #7 OR #8

#10 #1 AND #9
