# Supplementary material for: Metabolic Dysfunction–Associated Steatotic Liver Disease and Respiratory Disorders: A Systematic Review of Clinical and Pathophysiological Associations
Source: Curr Obes Rep. 2026 Apr 17;15(1):36. doi: 10.1007/s13679-026-00713-8 (PMC13090296; doi:10.1007/s13679-026-00713-8)
Supplement: Supplementary file 2 — Supplementary Material 2 [file 13679_2026_713_MOESM2_ESM.docx]

# **Supplementary Table S2. Data Extraction Form Template**

| **Study ID/ First Author/ Year/ Country** | **Study Design Setting /** | **Population Characteristics** | **Respiratory Disease Phenotype** | **Sample Size (n)** | **Mean/Median Age** | **Method for Assessing Hepatic Steatosis** | **Definition of Liver Disease (NAFLD/MAFLD/MASLD)** | **Liver Disease Severity (Fibrosis/Cirrhosis Assessment)** | **Respiratory Disease Diagnostic Criteria** | **Prevalence of Steatotic Liver Disease (%)** | **Main Respiratory Outcome** | **Effect Estimate (OR/RR/HR/β)** | **95% Confidence Interval** | **Variables Included in Adjusted Model** | **Key Findings** | **Funding Source** | **Conflicts of Interest** | **Notes** |
| --- | --- | --- | --- | --- | --- | --- | --- | --- | --- | --- | --- | --- | --- | --- | --- | --- | --- | --- |
|  |  |  |  |  |  |  |  |  |  |  |  |  |  |  |  |  |  |  |
|  |  |  |  |  |  |  |  |  |  |  |  |  |  |  |  |  |  |  |
|  |  |  |  |  |  |  |  |  |  |  |  |  |  |  |  |  |  |  |
|  |  |  |  |  |  |  |  |  |  |  |  |  |  |  |  |  |  |  |
|  |  |  |  |  |  |  |  |  |  |  |  |  |  |  |  |  |  |  |
|  |  |  |  |  |  |  |  |  |  |  |  |  |  |  |  |  |  |  |
|  |  |  |  |  |  |  |  |  |  |  |  |  |  |  |  |  |  |  |
|  |  |  |  |  |  |  |  |  |  |  |  |  |  |  |  |  |  |  |
