# Supplementary material for: Metabolic Dysfunction–Associated Steatotic Liver Disease and Respiratory Disorders: A Systematic Review of Clinical and Pathophysiological Associations
Source: Curr Obes Rep. 2026 Apr 17;15(1):36. doi: 10.1007/s13679-026-00713-8 (PMC13090296; doi:10.1007/s13679-026-00713-8)
Supplement: Supplementary file 3 — Supplementary Material 3 [file 13679_2026_713_MOESM3_ESM.docx]

# **Risk of bias assessment**

# **Newcastle–Ottawa Scale (NOS) Assessments – Cohort Studies**

| Study | Selection (max 4★) | Comparability (max 2★) | Outcome (max 3★) | Total |
| --- | --- | --- | --- | --- |
| Viglino et al. (2018) | ★★★ | ★ | ★★ | 6/9 |
| Tseng et al. (2025) | ★★★ | ★ | ★★ | 6/9 |
| Roh et al. (2022) | ★★★★ | ★★ | ★★★ | 9/9 |
| Chung et al. (2021) | ★★★★ | ★★ | ★★★ | 9/9 |
| Liu et al. (2025) | ★★★ | ★★ | ★★★ | 8/9 |
| Jördens et al. (2021) | ★★★★ | ★★ | ★★★ | 9/9 |
| Beibei et al. (2026) | ★★★★ | ★★ | ★★★ | 9/9 |
| Feng et al. (2024) | ★★★ | ★★ | ★★★ | 8/9 |
| Bocchino et al. (2015) | **★★★** | **★** | **★★** | 6/9 |
| Trzepizur et al. (2026) | **★★★★** | **★★** | **★★★** | 9/9 |

**Methodological Quality Assessment**

The methodological quality of the included analytical cross-sectional studies was evaluated using the Joanna Briggs Institute (JBI) Critical Appraisal Checklist for Analytical Cross-Sectional Studies, which includes eight items assessing selection criteria, exposure and outcome measurement, identification and management of confounding factors, and appropriateness of statistical analysis.

Although all studies fulfilled the reporting criteria of the JBI checklist, the overall risk of bias assessment also considered design-specific limitations inherent to cross-sectional research. These limitations include the absence of temporality between exposure and outcome, potential reverse causation, and susceptibility to residual confounding despite statistical adjustment.

For this reason, an additional interpretative framework was applied when determining the final risk of bias classification. Studies based on large nationally representative datasets with standardized measurements and robust multivariable modelling were classified as having low–moderate risk of bias, reflecting strong methodological execution despite inherent cross-sectional limitations. In contrast, studies conducted in clinic-based or highly selected populations, such as sleep clinic cohorts or bariatric surgery samples, were judged to have moderate risk of bias due to potential selection bias, reduced external validity, and increased likelihood of residual confounding.

Accordingly, no cross-sectional study was classified as unequivocally low risk of bias, given the intrinsic inability of this design to establish temporal relationships or causal inference.

| Study | Q1 | Q2 | Q3 | Q4 | Q5 | Q6 | Q7 | Q8 | Overall Risk of Bias |
| --- | --- | --- | --- | --- | --- | --- | --- | --- | --- |
| Viglino 2017 | Y | Y | Y | Y | Y | Y | Y | Y | Moderate |
| Lee 2020 | Y | Y | Y | Y | Y | Y | Y | Y | Low–Moderate |
| Zheng 2024 | Y | Y | Y | Y | Y | Y | Y | Y | Low–Moderate |
| Tsutsumi 2022 | Y | Y | Y | Y | Y | Y | Y | Y | Moderate |
| Moon 2018 | Y | Y | Y | Y | Y | Y | Y | Y | Low–Moderate |
| Sun 2025 | Y | Y | Y | Y | Y | Y | Y | Y | Low–Moderate |
| Benotti 2016/2018 | Y | Y | Y | Y | Y | Y | Y | Y | Moderate |
| Jawa 2021 | Y | Y | Y | Y | Y | Y | Y | Y | Moderate |
| Yu 2025 | Y | Y | Y | Y | Y | Y | Y | Y | Low–Moderate |
| Türker 2022 | Y | Y | Y | Y | Y | Y | Y | Y | Moderate |
| Jullian-Desayes 2021 | Y | Y | Y | Y | Y | Y | Y | Y | Moderate |

# **Quality Assessment of Mendelian Randomization Studies (STROBE‑MR)**

| **Study** | **Assessment Tool** | **Key Strengths** | **Key Limitations** | **Overall Risk of Bias** |
| --- | --- | --- | --- | --- |
| Yu 2025 | STROBE‑MR | Clear MR framework; genome‑wide significant SNP instruments; LD clumping; F‑statistics >10; multiple sensitivity analyses (IVW, MR‑Egger, weighted median, weighted mode); pleiotropy and heterogeneity testing (MR‑PRESSO, Cochran’s Q); leave‑one‑out analysis. | Exposure phenotype based on broad sleep apnea definition rather than polysomnography‑confirmed OSA; potential phenotype heterogeneity; possible sample overlap; association attenuated after MR‑PRESSO outlier removal. | Low–Moderate |

Abbreviations: MR = Mendelian Randomization; IVW = Inverse‑Variance Weighted; SNP = Single Nucleotide Polymorphism; LD = Linkage Disequilibrium; RoB = Risk of Bias.
